# Probiotic Modulation of the Gut–Ovary and Gut–Myometrium Axes: An In Vitro Study

**DOI:** 10.3390/microorganisms14030661

**Published:** 2026-03-14

**Authors:** Simone Mulè, Francesca Parini, Rebecca Galla, Francesca Uberti

**Affiliations:** 1 Laboratory of Physiology, Department for Sustainable Development and Ecological Transition, University of Piemonte Orientale, UPO, 13100 Vercelli, Italy; simone.mule@uniupo.it; 2 Noivita S.r.l.s., Spin Off of University of Piemonte Orientale, Strada Privata Curti n. 7, 28100 Novara, Italy; francescaparini00@gmail.com (F.P.); rebecca.galla@noivita.it (R.G.)

**Keywords:** gut–ovary axis, probiotics, myometrial function

## Abstract

Emerging evidence suggests that gut microbiota significantly influence female reproductive health by affecting hormonal, immune and metabolic processes. This research explored how a probiotic blend comprising *Lactobacillus crispatus* novaLCR6, *Limosilactobacillus fermentum* novaLF58 and *Bifidobacterium bifidum* novaBBF9 affects the gut–myometrium and gut–ovary axes. Intestinal epithelial cells were exposed to individual probiotics or their combination using a Transwell^®^ setup; their effects on barrier integrity, probiotic activity and short-chain fatty acid production were measured. Subsequently, basolateral metabolites were applied to myometrial and ovarian cells to assess viability, proliferation, oxidative stress, inflammation, signalling pathways and hormone production. All probiotics enhanced intestinal cell viability and barrier function. The combined probiotic showed synergistic effects, enhancing butyrate production by ~23–51%, improving myometrial proliferation by up to ~78%, decreasing ROS and TNF-α levels by ~49% and ~74% and modulating oxytocin signalling. In ovarian cells, the probiotic mixture activated ERK/MAPK and PI3K/AKT pathways, normalised PAK1, ERβ and PAX8 expressions and significantly increased LH and FSH secretion compared to single strains. These findings suggest that a multi-strain probiotic may modulate pathways involved in reproductive tissue homeostasis through gut–reproductive axis interactions, providing mechanistic insight from an *in vitro* study.

## 1. Introduction

Female fertility results from a multifaceted interplay among endocrine, metabolic, immunological and structural factors [1]. To achieve optimal conception and ensure successful pregnancy maintenance, it is essential to preserve ovarian and endocrine function, as well as the physiological integrity of the reproductive tissues, particularly the endometrium and myometrium [2]. Alterations in any of these compartments have been shown to significantly affect a female’s reproductive capacity [3,4].

The most prevalent disorders that impact fertility include polycystic ovary syndrome (PCOS) and alterations in myometrial trophism [5,6].

PCOS is a significant contributing factor to chronic anovulation and infertility, representing not only the most common endocrine-metabolic condition in women of childbearing age, but also one that is particularly amenable to treatment and management [7]. The condition is characterised by elevated androgens, irregular menstrual cycles, polycystic ovaries, chronic inflammation and alterations in glucose and lipid metabolism [8]. The aforementioned dysfunctions result in alterations in follicular growth, reduced oocyte quality, microenvironmental changes and a systemic inflammatory state, which collectively contribute to impairment of ovarian function and response to gonadotropic hormones [9,10,11]. Recent years have seen the addition of a new dimension to the understanding of the syndrome’s pathophysiology, with the roles of immune factors, intestinal dysbiosis and oxidative stress highlighted, alongside the endocrine and metabolic components. This has opened up new therapeutic perspectives, including the modulation of the microbiota [12,13,14].

About fertility, the trophism of the myometrium is also of significance. Myometrium is the smooth muscle tissue of the uterus, responsible for supporting and maintaining the integrity of the uterine cavity [15,16]. The endometrial tissue is considered healthy insofar as it provides a favourable environment for implantation, modulates muscle tone during the different phases of the cycle, and provides adequate support for pregnancy [17,18]. Consequently, alterations in trophism caused by inflammation, oxidative stress, or endocrine imbalances can have a detrimental effect on the uterine environment, thereby contributing to infertility [19]. It is therefore essential to understand the factors that modulate the myometrium, including the intestinal microbiota, which is gaining increasing interest in reproductive biology [20,21,22].

It is currently accepted that the intestinal microbiota is a pivotal regulator of the organism’s metabolic, endocrine and immune homeostasis [23,24]. In physiological conditions, a balanced microbiota has been shown to produce beneficial metabolites, including short-chain fatty acids (SCFAs) such as butyrate and propionate. These metabolites have been shown to support the intestinal barrier, modulate the inflammatory response and intervene in oestrogen metabolism [25,26]. In support of this, numerous studies highlight that marked intestinal dysbiosis, characterised by the release of *Lactobacillus* and *Bifidobacterium*, can be associated with chronic low-grade inflammation, increased intestinal permeability, interference with the regulation of sex hormone metabolism, and worsening of insulin resistance [27,28]. A considerable number of these phenomena have been identified as pathogenetic factors of PCOS, and preliminary clinical studies have demonstrated that modulation of the intestinal microbiota, through the administration of probiotics or prebiotics, can enhance hyperandrogenism, metabolic profile and ovarian inflammation [29,30,31]. For instance, in an animal model of PCOS, supplementation with a butyric acid analogue has been shown to improve the menstrual cycle, normalise the hormonal profile, restore a physiological ovarian morphology and regulate the expression of steroidogenesis factors in granulosa cells [31]. However, in human ovarian cell cultures, the incorporation of butyric acid has been shown to reduce the expression of inflammatory cytokines such as interleukin 6 (IL-6), and Tumour Necrosis Factor α (TNF-α) and inflammasome components like NLR Family Pyrin Domain Containing 3 (NLRP3), indicating an anti-inflammatory effect [32].

Preliminary experimental and clinical studies suggest that specific probiotic strains belonging to the genera *Lactobacillus* and *Bifidobacterium* can positively influence ovarian function. A study in mice and rats, in which the animals were treated with letrozole to induce PCOS, demonstrated that administration of selected lactobacilli or bifidobacteria attenuated the ovarian changes characteristic of this syndrome. Indeed, a normalisation of ovarian morphology, a reduction in hormonal imbalances (Luteinising Hormone (LH), Follicle-Stimulating Hormone (FSH), testosterone), and an increase in intestinal levels of SCFAs, associated with an improved endocrine profile, were observed [33]. In a clinical study of women diagnosed with PCOS, the administration of *Bifidobacterium lactis* V9 for weeks resulted in alterations to the composition of the intestinal microbiota, an increase in faecal SCFA, and a significant reduction in LH and the LH/FSH ratio in a proportion of participants. These findings suggest that the modulation of the microbiota may be reflected in hormonal parameters relevant to ovarian function [34,35]. Moreover, a recent review and meta-analysis of probiotic interventions in women with PCOS highlighted that supplementation with *Lactobacillus* and *Bifidobacterium* strains, either individually or in combination, is associated with improvements in metabolic and hormonal parameters, including increased sex hormone-binding globulin (SHBG), reduced insulin resistance and decreased markers of oxidative stress and inflammation [36,37,38]. These findings lend support to the hypothesis that specific probiotics may modulate ovarian function by regulating the gut microbiota, with potential benefits for female reproductive health.

In this context, probiotics emerge as potential modulators of reproductive health [39,40]. According to the definitions established by the Food and Agriculture Organisation of the United Nations (FAO) and the World Health Organisation (WHO), these are live microorganisms that, when administered in adequate amounts, confer benefits to the host [41]. Species such as *Lactobacillus crispatus*, *Lactobacillus fermentum* and *Bifidobacterium bifidum* have been the focus of significant research efforts, particularly regarding their capacity to enhance microbiota homeostasis, fortify the intestinal and vaginal barriers, generate lactic acid and other bioactive metabolites, and regulate the immune response [42,43,44,45].

These findings suggest a possible role for probiotics in supporting both ovarian function in PCOS and myometrial trophism via the gut–ovary and gut–myometrium axes. Consequently, this study aims to analyse the properties of specific probiotics, namely *Lactobacillus crispatus*, *Lactobacillus fermentum* and *Bifidobacterium bifidum*, in relation to female fertility. The objective of the present study was twofold: firstly, to investigate the potential role of these strains in supporting myometrial trophism and function in vitro; and secondly, to examine their possible modulatory action on ovarian physiology and the characteristic mechanisms of PCOS in vitro. These investigations were conducted in an in vitro setting, in which an in vitro model of gut–target tissue communication was established to simulate metabolite-mediated interactions between intestinal cells and reproductive tissues.

## 2. Materials and Methods

### 2.1. Sample Preparation

*Lactobacillus crispatus* novaLCR6 DSM 34348 (*L. crispatus* novaLCR6), *Limosilactobacillus fermentum* novaLF58 DSM 34340 (*L. fermentum* novaLF58) and *Bifidobacterium bifidum* novaBBF9 DSM 34337 (*B. bifidum* novaBBF9) lyophilised powders were provided by Probionova (Lugano, Switzerland). All strains are registered at the Deutsche Sammlung von Mikroorganismen (DSMZ), ensuring taxonomic validation and traceability. These strains were selected based on well-defined scientific criteria, considering characteristics reported in the literature (i) their documented role in maintaining intestinal and vaginal microbiota homeostasis [46]; (ii) their capacity to produce bioactive metabolites, particularly short-chain fatty acids [47]; (iii) evidence supporting anti-inflammatory and immunomodulatory properties [48]; and (iv) emerging data suggesting potential endocrine-modulating effects relevant to ovarian physiology and PCOS-related alterations [49]. The lyophilised preparations were dissolved directly in Phosphate-buffered saline 1x (PBS, Merck Life Science, Rome, Italy) to prepare several concentrations for testing. *L. crispatus* novaLCR6, *L. fermentum* novaLF58 and *B. bifidum* novaBBF9 were tested in a range from 5 to 50 mg/mL (corresponding to a range of 0.5 × 10^9^ and 5 × 10^9^ CFU) [50,51,52]. The conversion from Colony Forming Unit (CFU) to mg is reported in Table 1 for each probiotic strain. Because of differences in CFU-to-mg conversion among strains, concentration selection was based on functional biological efficacy rather than identical mg amounts, ensuring comparable biological activity. Before each stimulation, a new pack of the probiotics was mixed and reconstituted into Dulbecco’s Modified Eagle’s Medium (DMEM; GIBCO^®^ ThermoFisher Scientific, Waltham, MA, USA) without red phenol, supplemented with 0% Foetal Bovine Serum (FBS; Merck Life Science, Rome, Italy), 1% penicillin–streptomycin and 2 mM L-glutamine solution (all from Merck Life Science, Rome, Italy). The samples dissolved in PBS1x were diluted directly into the culture medium in the well to obtain the final concentration.

### 2.2. Cell Culture

The human epithelial intestinal cells, Caco-2, purchased from the American Type Culture Collection (ATCC, Manassas, VA, USA), were cultured in Dulbecco’s Modified Eagle’s Medium/Nutrient F-12 Ham’s (DMEM-F12, GIBCO^®^ ThermoFisher Scientific, Waltham, MA, USA) containing 10% FBS, 2 mM L-glutamine and 1% penicillin–streptomycin (all from Merck Life Science, Rome, Italy) and maintained in an incubator at 37 °C and 5% CO_2_ [53]. The cells used in the experiments were at passage numbers between 26 and 32 to preserve paracellular permeability and transport properties, thereby maintaining similarity to the intestinal absorption mechanism following oral intake in humans [54]. The Food and Drug Administration (FDA) and the European Medicines Agency (EMA) have authorised the use of this cell line in experimental models to predict drug absorption, metabolism and bioavailability after oral administration [55,56]. Specifically, for this model, 6.5 mm Transwell^®^ inserts and a polycarbonate membrane with 0.4 μm pores were used to plate 2 × 10^4^ cells in a 24-well plate. Before stimulation, the cells were incubated for 8 h in an incubator in DMEM medium without red phenol, supplemented with 1% penicillin–streptomycin, 0.5% FBS and 2 mM L-glutamine (all from Merck Life Science, Rome, Italy) [54].

The PHM1-41 cell line (ATCC, Manassas, VA, USA) was derived from pregnant human myometrium and was maintained in DMEM (GIBCO^®^ ThermoFisher Scientific, Waltham, MA, USA) supplemented with 10% FBS, 0.1 mg/mL gentamicin (G-418), 2 mM glutamine (all from Merck Life Science, Rome, Italy) and were incubated at 37 °C, 5% CO_2_ and 95% humidity [53]. For the experiments, 5 × 10^4^ cells were seeded on the bottom of a 24-well plate containing the Transwell^®^ system with Caco-2 cells.

The COV434 line of immortalised granulosa cells was derived from a primary human granulosa cell tumour and was purchased by AcceGen (Fairfield, Fairfield Rd, NJ, USA). COV434 cells were cultivated in DMEM–F12 with GlutaMAX™ (GIBCO^®^ ThermoFisher Scientific, Waltham, MA, USA), with the addition of 9% FBS, henceforth referred to as complete media, at 37 °C in a humidified atmosphere containing 95% air and 5% CO_2_. The cells utilised in the experiments documented herein were derived from passages 41–77. The standard procedure for harvesting cells involved the use of trypsinisation with 0.25% trypsin–EDTA, followed by counting using a haemocytometer and 0.4% trypan blue. The cells were then plated in complete media or complete media supplemented with 1 mg/mL G418 [57]. For the experiments, 7 × 10^5^ cells were seeded on the bottom of a 24-well plate containing the Transwell^®^ system with Caco-2 cells.

### 2.3. Experimental Protocol

**Figure 1:**
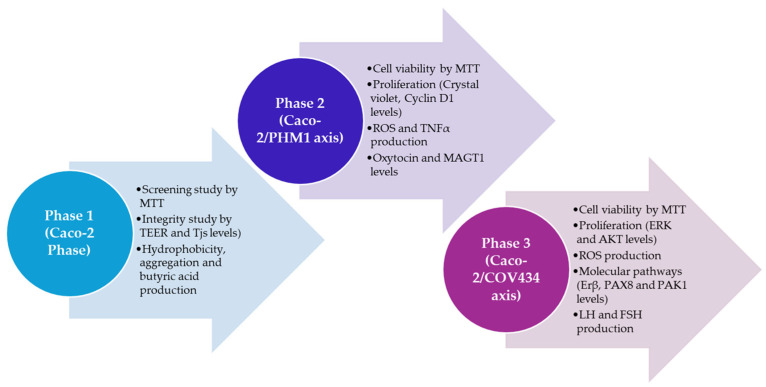
Schematic experimental protocol. Each phase and method used was represented.

The study was divided into three phases (Figure 1). The first phase was designed to evaluate the effects of different probiotic strains at the intestinal level. The second phase was designed to evaluate the effects of their metabolites on target tissues, such as the ovary and myometrium. These target tissues were generated in vitro.

Phase 1. In the initial phase, the study focused on identifying the concentration of each probiotic strain to be utilised. Consequently, a series of time-dependent dose–response screening tests was conducted on Caco-2 intestinal cells over 1 h to 6 h. The purpose of these tests was to evaluate the viability of intestinal cells following treatment with varying concentrations of the probiotic strains under investigation. The viability of the intestinal cells was determined using an MTT test. The MTT assay was employed not only to ensure the absence of cytotoxicity but also to provide insight into the potential proliferative effects of microbial metabolites on host cells. The MIX (novaFERT^®^) was subsequently formulated by combining the three probiotic strains at their individually determined optimal concentrations, as identified from the MTT screening tests. Subsequently, once the optimal concentration for each probiotic strain had been determined, the intestinal cells were seeded in Transwell^®^ media to assess whether the probiotic strains at the selected concentration and their combination (novaFERT^®^, in the manuscript called MIX, was composed by *L. crispatus* novaLCR6 10 mg/mL, *L. fermentum* novaLF58 5 mg/mL and *B. bifidum* novaBBF9 10 mg/mL) adhered to the intestinal epithelium and produced short-chain fatty acids that crossed the intestinal barrier and reached the bloodstream. Specifically, the integrity of the cell monolayer was analysed by measuring the Transepithelial Electrical Resistance (TEER) and by evaluating the levels of tight junction (TJ) proteins, the concentration of butyric acid that was able to cross the intestinal barrier (using an ELISA kit; Cloud-Clone, Wuhan, China), and the ability of the probiotics to aggregate and hydrophobicity using specific tests (aggregation and hydrophobicity tests). Aggregation and hydrophobicity assays were included to assess the strains’ potential adhesion to the intestinal epithelium, a prerequisite for effective metabolite delivery to downstream tissue.

Phase 2. In the subsequent phase, a gut–myometrium axis was reconstructed using the Transwell^®^ system. Specifically, Caco-2 intestinal cells were seeded in the apical compartment of the Transwell^®^ insert, while PHM1-41 myometrial cells were placed in the basolateral compartment. Following treatment at the apical level with the selected probiotic strains, the gut–myometrium axis was incubated for 24 h. Thereafter, all processes involved in maintaining myometrial trophism were investigated in PHM1-41 cells. Consequently, at the conclusion of the stimulation, the following analyses were conducted: cell viability assessment utilising the MTT test, cell proliferation evaluation employing Crystal violet staining, and cyclin D1 level analysis. Additionally, oxidative stress (evaluating the reduction of cytochrome C; Merck Life Science, Rome, Italy), inflammatory state (assessing TNF-α using the ELISA Kit; Merck Life Science, Rome, Italy), and the contractile activity of myometrial cells (evaluating oxytocin and magnesium transport 1, MAGT1, levels) were analysed.

Phase 3. In this final phase, an intestine–ovary axis was reconstructed using the Transwell^®^ system. Specifically, Caco-2 intestinal cells were seeded in the apical portion of the Transwell^®^ insert, while COV434 ovarian cells were placed at the basolateral level. Following treatment at the apical level with the selected probiotic strains, the intestine–ovary axis was incubated for 24 h. Thereafter, all processes involved in maintaining ovarian function were investigated in COV434 cells. Consequently, at the conclusion of the stimulation, the following parameters were analysed: cell viability, employing the MTT test, and oxidative stress by reducing Cytochrome C, cell proliferation by analysing ERK and AKT levels, the mechanisms implicated in ovarian dysfunction in PCOS such as Erβ, PAX8, and PAK-1, and the hormone levels of LH and FSH using a specific ELISA kit.

During the analyses, the cells were maintained in DMEM without red phenol and 0.5% FBS (GIBCO^®^ ThermoFisher Scientific, Waltham, MA, USA), 2 mM L-glutamine, and 1% penicillin–streptomycin (Merck Life Science, Rome, Italy).

### 2.4. Intestinal Barrier In Vitro Model

In accordance with the literature [58] and the standards set by the EMA and FDA [55,56], an in vitro intestinal barrier model was developed using the Transwell^®^ system to estimate the absorption, metabolism and bioavailability of several substances after oral intake in humans.

The initiation of this experiment involved seeding Caco-2 cells according to established protocols. The cells were then cultured in complete growth medium for 21 days. During this period, the culture medium was systematically transferred between the basolateral and apical compartments to ensure optimal growth conditions and promote proper cellular differentiation. On the 21st day, when TEER values were ≥400 Ω·cm^2^ [59], butyric acid quantification and adhesion of probiotic strains to intestinal epithelium were performed from 1 to 6 h. Before stimulation, the medium was adjusted to pH 6.5, which corresponds to the pH of the small intestinal lumen. In addition, the pH on the basolateral side was adjusted to 7.4, representing the pH of blood [59]. To ensure the levels had stabilised adequately, the cells were maintained at 37 °C in an atmosphere containing 5% CO_2_ for 15 min before the experiment began.

### 2.5. MTT Test

After each stimulation, cells were washed with PBS1x and incubated with DMEM without red phenol and FBS containing 1% MTT dye (MTT-Based In Vitro Toxicology Assay Kit; Sigma-Aldrich, Saint Louis, MO, USA) for 2 h at 37 °C and 5% CO_2_ [58]. Cell viability was determined by measuring absorbance at 570 nm with a correction at 690 nm using a spectrophotometer (Infinite 200 Pro MPlex, Tecan, Männedorf, Switzerland). It was expressed as the percentage mean (%) ± standard deviation (SD) relative to the control (0% viable) across five independent experiments conducted in triplicate.

### 2.6. Butyric Acid Quantification

To quantify probiotic metabolites, butyric acid production was analysed using an ELISA kit (Cloud-Clone, Wuhan, China) according to the manufacturer’s instructions [60]. The absorbance of each sample was measured after the addition of stop solution at 450 nm using a plate reader (Infinite 200 Pro MPlex, Tecan, Männedorf, Switzerland) and the OD were interpolarised with a standard curve (from 0 to 10,000 pg/mL), expressing the data as mean (%) ± SD compared to control of five different tests, each carried out in triplicate.

### 2.7. Surface Hydrophobicity and Aggregation Assay

The surface hydrophobicity and aggregation assay methods were conducted in accordance with the established methodology outlined in the literature [61]. The present study utilised PBS as a control, whilst 0.4 mL of xylene was mixed with 4 mL of a probiotics-containing suspension. This was then left to stand for a period of 15 min in order to yield the aqueous phase. The absorption values for the sample and control were measured at 600 nm. Each analysis was conducted thrice, utilising ten parallel samples. The surface hydrophobicity of each sample in this study was then calculated according to Equation (1):

(1) $$\mathrm{Surface}\;\mathrm{Hydrophobicity}\;=\;\frac{{OD}_{600}(control)-{OD}_{600}(test)}{{OD}_{600}(control)}\times \;100\%$$

where OD_600_ (control) represents the absorbance of the control suspension and OD_600_ (test) represents the absorbance after xylene treatment.

Subsequently, 0.1 mL of probiotic suspension was added to 2.9 mL of PBS, and the absorbance was measured at 600 nm. Aggregation activity was calculated according to Equation (2):

(2) $$\mathrm{Aggregation}\;\mathrm{Activity}\;=\;\frac{{OD}_{600}(0)-{OD}_{600}(t)}{{OD}_{600}(0)}\times \;100\%$$

where OD_600_ (0) represents the absorbance immediately after suspension preparation, and OD_600_ (t) represents the absorbance after 2 h.

### 2.8. Gut/Myometrium Co-Culture Model

The Caco-2 cells were seeded into Transwell^®^ inserts at a density of 20,000 cells/insert, as previously described [58], and were grown for 21 days in a complete medium, as previously reported. Subsequently, a period of 3–5 days preceded the designated deadline, during which the PHM1-41 cells were introduced to the basolateral side of the insert system at a concentration of 5 × 10^4^ cells. The co-culture was then maintained in complete medium for 3 days. The medium was changed from the apical side of the wells, and the samples were then incubated for up to two hours. Subsequently, treatments were performed with the probiotic strains under study at the selected concentration, alone and in combination, added apically and incubated for 24 h. This incubation period was sufficient to allow probiotics to produce short-chain fatty acids, which have been shown to influence myometrial cells. Following the conclusion of the experiment, the Transwell^®^ insert was removed to conduct subsequent tests on PHM1-41 cells. These included analyses of cell viability, oxidative stress, the inflammatory process, the cell cycle, contraction mechanisms and the intracellular mechanisms involved in the myometrial trophism.

### 2.9. Gut/Ovarian Co-Culture Model

Three days before the completion of the gut cell maturation process, 7 × 10^5^ COV434 cells were seeded into wells of a 24-well plate and maintained at 37 °C until a cell monolayer was formed. Following a three-day maturation period for both gut and ovarian cells, the Transwell^®^ insert containing the gut cells was placed in the 24-well plate seeded with COV434 cells. Subsequently, the probiotic strains under study were administered at their respective concentrations, both individually and in combination, via the apical route, and the samples were then incubated for 24 h. Following the conclusion of the experiment, the Transwell^®^ insert was removed to conduct subsequent tests on COV434 cells. These encompassed analyses of cell viability, oxidative stress, mechanisms implicated in cell proliferation, mechanisms involved in ovarian dysfunction in PCOS, and LH and FSH levels.

### 2.10. Crystal Violet

Cell proliferation in PHM1-41 cell lysates was assessed by Crystal violet staining, as reported in the literature [58]. After treatment, the cells were washed with PBS 1× and fixed with 1% glutaraldehyde in PBS 1× (% *v*/*v*) for 15 min at room temperature. 100 µL of 0.1% Crystal violet in aqueous solution (% *v*/*v*) was added to each well for 20 min at room temperature. The cells were washed again, and 100 µL of 10% acetic acid in PBS (1× % *v*/*v*) was added to solubilise them, and the absorbance was read on a spectrophotometer (Infinite 200 Pro MPlex, Tecan, Männedorf, Switzerland) at 595 nm. The number of cells was calculated by comparing data to control cells and normalised to untreated cells examined at time zero. The results were expressed as the mean (%) ± SD of five independent experiments conducted in triplicate.

### 2.11. ROS Production

The rate of superoxide anion release was measured in PHM1-41 cell supernatants using a standard protocol based on the reduction of cytochrome C [53]. In both treated and untreated cells, 100 μL of cytochrome C was added, and in another sample, 100 μL of superoxide dismutase was added for 30 min in an incubator (all substances were from Sigma-Aldrich). Absorbance in the culture supernatants was measured at 550 nm using a spectrometer (Infinite 200 Pro MPlex, Tecan, Männedorf, Switzerland), and O_2_ was expressed as the mean (%) ± SD of nanomoles of reduced cytochrome C per microgram of protein vs. control, based on five independent experiments conducted in triplicate.

### 2.12. Cyclin D1 ELISA Kit

Cyclin D1 production in PHM1-41 cell lysates was determined using Human Cyclin D1 (CCND1) ELISA kit (MyBiosource, San Diego, CA, USA), analysing cyclin D1 in PHM1-41 cell lysates, according to the manufacturer’s instructions. Briefly, 100 µL of each sample was added to the well, and the plate was incubated for 80 min at 37 °C. Afterwards, the wells were washed 3 times with 200 µL of Wash Solution, and 100 µL of Biotinylated Antibody was added before incubating the plate for 50 min at 37 °C. Then the wells were washed again for 3 times with 200 µL of Wash Solution before adding 100 µL of Streptavidin–HRP working solution for 50 min at 37 °C. Before adding 90 µL of TMB Substrate Solution to each well, the wells were washed again for 5 times. Finally, after 50 min of incubation at 37 °C, 50 µL of Stop reagent was added to each well. The samples were analysed by a spectrometer (Infinite 200 Pro MPlex, Tecan, Männedorf, Switzerland) at 450 nm. The concentration is expressed as ng/mL relative to a standard curve (range from 0.32 to 20 ng/mL), and the results are expressed as percentage mean (%) ± SD relative to the control (0% line) from five independent experiments conducted in triplicate.

### 2.13. TNF-α ELISA Kit

TNF-α concentration in PHM1-41 cell lysates was determined using the TNF-α ELISA kit (Merck Life Science, Rome, Italy) according to the manufacturer’s instructions. The absorbance of each well was measured at 450 nm after the addition of the stop solution using a plate reader (Infinite 200 Pro MPlex, Tecan, Männedorf, Switzerland) [62]. The results were presented as percentage mean (%) ± SD versus the control (0 line) of five independent experiments conducted in triplicate.

### 2.14. Oxytocin ELISA Kit

The Oxytocin (OT) concentration in PHM1-41 cell lysates was determined by Oxytocin ELISA Kit (Cayman Chemical Company; Ann Arbor, MI, USA) according to the instructions. This assay is based on the competition between oxytocin and Oxytocin acetylcholinesterase (AChE) conjugated (Oxytocyn Tracer) for a limited amount of Oxytocin Polyclonal Antiserum. Because the concentration of the Oxytocin Tracer is held constant while the concentration of oxytocin varies, the amount of Oxytocin Tracer that can bind to the Oxytocin Polyclonal Antiserum will be inversely proportional to the concentration of oxytocin in the well. The plate was washed to remove any unbound reagents, and then Ellman’s Reagent was added to the wells. The product of the enzymatic reaction was read at wavelengths between 405 and 420 nm using a spectrophotometer (Infinite 200 Pro MPlex, Tecan, Männedorf, Switzerland). A standard curve is plotted relating the colour intensity to the concentration of the standard (range: 15.625–1000 pg/mL). The results were presented as the mean percentage (%) ± SD, compared with the control (0 line), from five independent experiments conducted in triplicate.

### 2.15. MAGT1 ELISA Kit

MAGT1 levels were determined using Magnesium Transporter Protein 1 (MAGT1) ELISA Kit (MyBioSource, Vancouver, BC, Canada) that analysed MAGT1 in PHM1-41 cell lysates, according to the manufacturer’s instructions. Briefly, 100 µL of each sample was added to the well, and the plate was incubated for 80 min at 37 °C. Afterwards, the wells were washed 3 times with 200 µL of Wash Solution, and 100 µL of Biotinylated Antibody was added before incubating the plate for 50 min at 37 °C. Then the wells were washed again for 3 times with 200 µL of Wash Solution before adding 100 µL of Streptavidin–HRP working solution for 50 min at 37 °C. Before adding 90 µL of TMB Substrate Solution to each well, the wells were washed again for 5 times. Finally, after 50 min of incubation at 37 °C, 50 µL of Stop reagent was added to each well. The samples were analysed by a spectrometer (Infinite 200 Pro MPlex, Tecan, Männedorf, Switzerland) at 450 nm. The concentration is expressed as ng/mL relative to a standard curve (range from 0.25 ng/mL to 8 ng/mL), and the results are expressed as percentage mean (%) ± SD relative to the control (0% line) from five independent experiments conducted in triplicate.

### 2.16. ERK Activity Assay

The activity of the ERK/MAPK signalling pathway in COV434 cell lysates was measured by a specific kit (InstantOne™; from Thermo Fisher, Milan, Italy) [63], following the manufacturer’s instructions. In summary, 50 μL/well of astrocyte lysate prepared with cell lysis buffer was tested in InstantOne™ ELISA microplate strips after a one-hour incubation at room temperature on a microplate shaker with the antibody cocktail. Subsequently, the detection reagent was added for 20 min, after which the stop solution was added to halt the process. Using a spectrometer (Infinite 200 Pro MPlex, Tecan, Männedorf, Switzerland), the absorbance was measured at 450 nm, and the results were expressed as the mean percentage (%) ± SD relative to the control (0 line) from five separate tests performed in triplicate.

### 2.17. ELISA Kit Quantification

Quantification of phosphorylated AKT (phospho) [pS473] and ERβ in COV434 cell lysates was performed using commercial ELISA kits according to the manufacturers’ instructions [63,64].

Also, PAX8 and PAK1 were measured using ELISA kits (MyBiosource, San Diego, CA, USA) according to the manufacturer’s instructions, while LH and FSH were measured using an ELISA kit from FineTest (Wuhan, China). Briefly, 100 µL of each sample or standard was added to the wells and incubated at 37 °C for 1 h, followed by washes and incubation with HRP–Streptavidin conjugate. After additional washes, TMB substrate was added, the reaction was incubated, the reaction was stopped with Stop Solution, and absorbance was measured at 450 nm using a spectrometer (Infinite 200 Pro MPlex, Tecan, Männedorf, Switzerland). The concentrations were calculated and compared to kit-specific standard curves (AKT [pS473]: 1.6–50 U/mL; ERβ: 0.312–20 ng/mL; PAX8: 31.2–1000 pg/mL; PAK1: 10–3000 ng/L); LH: 0.938–60 mIU/mL; and FSH: 1.563–100 mIU/mL. Results are expressed as mean ± SD percentage relative to control (0 line) from five independent experiments performed in triplicate.

### 2.18. Statistical Analysis

Results are expressed as mean percentage (%) ± SD of at least 5 biological replicates for each experimental protocol, and each replicate was replicated 3 times for each experimental protocol. Statistical comparisons between groups were performed using one-way ANOVA with Bonferroni’s post hoc test or the Mann–Whitney U test, as appropriate, using GraphPad Prism 5 (GraphPad Software, La Jolla, CA, USA). *p* < 0.05 was considered statistically significant. All densitometric analysis data were normalised to control values (defined as 1). All other data from each experimental protocol were normalised to the control values as a percentage (defined as 0%).

## 3. Results

### 3.1. Concentration Screening of Probiotic Strains Under Study on Intestinal Cells

**Figure 2:**
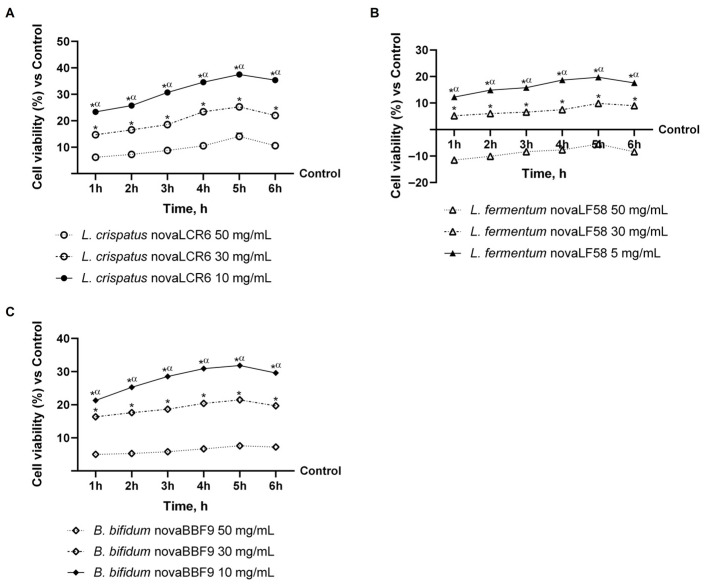
Concentration screening of probiotic strains under study on intestinal cells after treatment for 1 to 6 h. In (**A**), cell viability analysis of *L. crispatus* novaLCR6 was measured by the MTT test; in (**B**), cell viability analysis of *L. fermentum* novaLF58 was measured by the MTT test; and in (**C**), cell viability analysis of *B. bifidum* novaBBF9 was measured by the MTT test. Data are mean ± SD of five independent experiments performed in triplicate compared to the control (0% line). *p* < 0.05 vs. control; * *p* < 0.05 vs. 50 mg/mL concentration; α *p* < 0.05 vs. 30 mg/mL concentrations.

In the first part of the study, experiments were performed to determine the best concentration for each probiotic strain studied on intestinal cells. Specifically, the probiotic strains *L. crispatus* novaLCR6, *L. fermentum* novaLF58 and *B. bifidum* novaBBF9 were tested in a concentration range of 5–50 mg/mL (corresponding to a range of 0.5 × 10^9^ and 5 × 10^9^ CFU) and analysed through the MTT test. As illustrated in Figure 2A, the cell viability data were obtained following stimulation with *L. crispatus* novaLCR6 over a time interval ranging from 1 to 6 h and within a concentration range of 10–50 mg/mL. All examined concentrations significantly increased viability compared to the control (untreated cells; *p* < 0.05). Peak activity was consistently observed after five hours of treatment, with viability significantly higher than that of the control group (*p* < 0.05). Among these, the 10 mg/mL concentration was found to be the most effective: after 5 h of treatment, it exceeded the effect of the 30 mg/mL concentration by approximately 33% (*p* < 0.05) and that of the 50 mg/mL concentration by approximately 62.5% (*p* < 0.05).

Furthermore, Figure 2B shows the effects on intestinal cell viability following treatment with *L. fermentum* novaLF58 over the stimulation range of 1 to 6 h. The findings, derived from analysis of the data, indicated that two of the three concentrations of the probiotic strain *L. fermentum* novaLF58 examined enhanced cell viability compared with the untreated control (5 mg/mL and 30 mg/mL; *p* < 0.05 vs. control). In addition, the maximal effect was discerned following a 5 h period of treatment, with the 5 mg/mL concentration exhibiting a substantially augmented effect, marked by an approximate 50% increase compared to the 30 mg/mL concentration (*p* < 0.05) and by an approximate 1.28-fold increase compared to the 50 mg/mL concentration (*p* < 0.05). 

Finally, Figure 2C presents the results of cell viability analysis following treatment with *B. bifidum* novaBBF9 over the treatment period of 1 to 6 h at concentrations ranging from 10 to 50 mg/mL. Once more, it was observed that all concentrations enhanced cell viability compared to the control group (*p* < 0.05). However, the most significant increase in cell viability was recorded after 5 h of treatment with *B. bifidum* novaBBF9 at a concentration of 10 mg/mL, in comparison to concentrations of 30 mg/mL (approximately 32.5%, *p* < 0.05) and 50 mg/mL (approximately 76.1%, *p* < 0.05).

In conclusion, the present findings indicated that *L. crispatus* novaLCR6, *L. fermentum* novaLF58 and *B. bifidum* novaBBF9 may significantly enhance cell viability, with optimal efficacy observed for all strains after a five-hour incubation period. In addition, for all probiotic strains examined in this study, the concentration demonstrating optimal performance was 10 mg/mL (*L. crispatus* novaLCR6 and *B. bifidum* novaBBF9) or 5 mg/mL (*L. fermentum* novaLF58), and this concentration will be utilised in subsequent experimental investigations.

**Figure 3:**
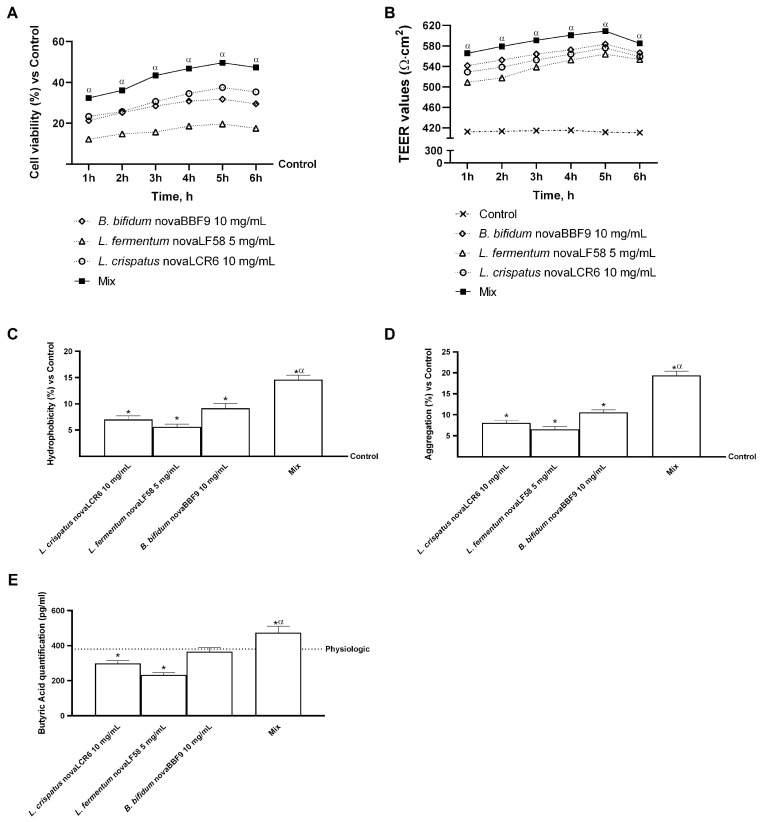
Intestinal barrier analysis. In (**A**), cell viability analysis was assessed by MTT at 1 h to 6 h; in (**B**), TEER analysis was performed from 1 h to 6 h; in (**C**), surface hydrophobicity of probiotic strains was determined after 6 h of treatment using the xylene partitioning method, where higher hydrophobicity indicates greater potential for epithelial adhesion; in (**D**) auto-aggregation of probiotic strains was assessed after 6 h by monitoring the decrease in optical density at 600 nm over time, as a measure of strain aggregation ability; and in (**E**), butyric acid quantification was measured at 6 h of treatment. Mix = *L. crispatus* novaLCR6 10 mg/mL + *L. fermentum* novaLF58 5 mg/mL + *B. bifidum* novaBBF9 10 mg/mL. Data are expressed as mean ± SD (%) of 5 independent experiments performed in triplicate, normalised to the control. In (**A**,**B**), *p* < 0.05 vs. control; α *p* < 0.05 vs. single probiotic strains. In (**C**–**E**), * *p* < 0.05 vs. control; α *p* < 0.05 vs. single probiotic strains.

A series of experiments was conducted on the Transwell^®^ intestinal model to evaluate several parameters, including cell viability, cell monolayer integrity, hydrophobicity, and the probiotics’ aggregation capacity. Additionally, the production of short-chain fatty acids, particularly butyric acid, was examined following treatment with probiotic strains in isolation and in combination. As demonstrated in Figure 3A, all individual probiotic strains exhibited a capacity to enhance cell viability in comparison to the control group (*p* < 0.05), reaching a maximum at the 5 h treatment point, with approximate increases of 36% (*B. bifidum* novaBBF9 vs. control, *p* < 0.05), 60% (*L. fermentum* novaLF58 vs. control, *p* < 0.05) and 24% (*L. crispatus* novaLCR6 vs. control, *p* < 0.05). Notably, the combination of strains further amplified this effect, with a peak at 5 h of treatment compared with *L. crispatus* novaLCR6, *L. fermentum* novaLF58 and *B. bifidum* novaBBF9 (*p* < 0.05). To corroborate these findings, TEER levels were meticulously monitored throughout the treatment interval to ensure the integrity of the cell monolayer (Figure 3B). Following the combination, the probiotic strains suggested an augmented effect in comparison to the individual agents and the control group (*p* < 0.05). Additionally, the hydrophobicity, aggregation capacity of the probiotics, and butyrate production at the intestinal monolayer level were evaluated. As shown in Figure 3C,D, all probiotic strains exhibited a hydrophobic membrane and a high aggregation capacity compared to the control (*p* < 0.05). The data presented in Figure 3C reveal that the combination treatment increased the level of hydrophobicity by approximately 37%, 62% and 52% compared to *B. bifidum* novaBBF9, *L. fermentum* novaLF58 and *L. crispatus* novaLCR6, respectively (*p* <0.05). Similarly, Figure 3D shows that the aggregation capacity of the mixture increased by approximately 45%, 66% and 58% compared to *B. bifidum* novaBBF9 (*p* < 0.05), *L. fermentum* novaLF58 (*p* < 0.05) and *L. crispatus* novaLCR6 (*p* < 0.05), respectively. 

In conclusion, Figure 3E shows the quantification analysis of butyric acid produced by probiotics that were able to cross the intestinal barrier throughout the treatment interval. Compared to the physiological production of butyric acid, *L. crispatus* novaLCR6 and *L. fermentum* novaLF58 showed a reduced production of butyric acid (*p* < 0.05), while *B. bifidum* novaBBF9 equalled the physiological production of butyric acid at the intestinal level after 6 h of treatment. Treatment with MIX markedly enhanced butyrate production with an increase of approximately 37% (vs. *L. crispatus* novaLCR6, *p* < 0.05), 51% (vs. *L. fermentum* novaLF58, *p* < 0.05) and 23% (vs. *B. bifidum* novaBBF9, *p* < 0.05).

### 3.2. Analysis of the Effects of Probiotics on the Gut–Myometrium Axis

At the end of the 6 h treatment period, a quantity of probiotic metabolites was released into the basolateral environment of the Transwell^®^ model (gut–myometrium axis). These were then analysed for their ability to modulate the trophism of myometrial cells (PHM-1). In the initial phase of the experiment, a range of tests were carried out to assess cell viability, proliferation, radical oxygen species (ROS) production and TNF-α levels in myometrial cells. 

**Figure 4:**
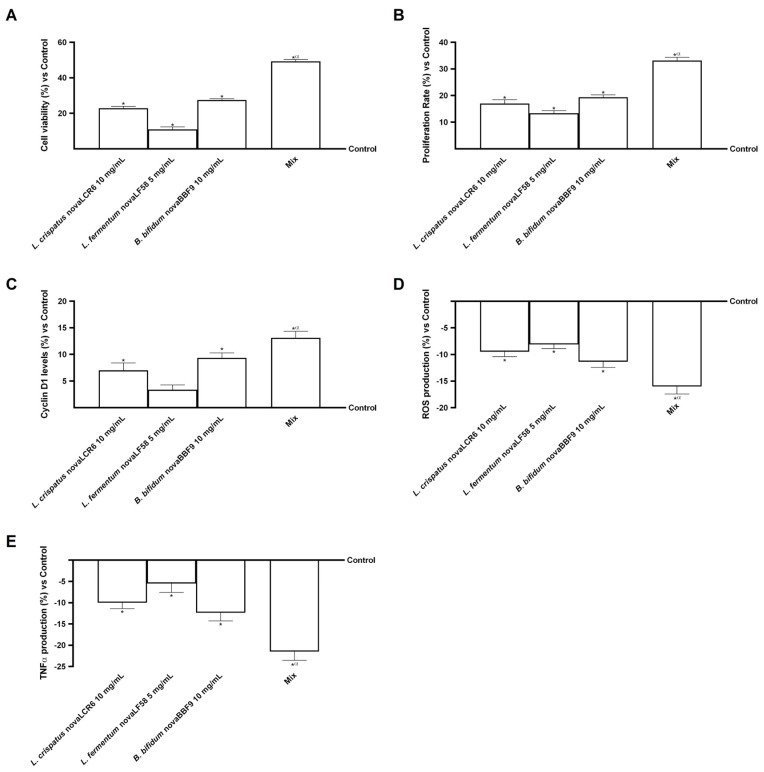
Proliferation analysis in myometrial cells. In (**A**), cell viability analysis was assessed by the MTT test; in (**B**), proliferation analysis was assessed by crystal violet staining; in (**C**), Cyclin D1 activity was assessed by the ELISA kit; in (**D**), ROS production was measured by Cytochrome C reduction; and in (**E**), TNFα production was analysed by an ELISA kit. Mix = *L. crispatus* novaLCR6 10 mg/mL + *L. fermentum* novaLF58 5 mg/mL + *B. bifidum* novaBBF9 10 mg/mL. Data are expressed as mean ± SD (%) of 5 independent experiments, normalised and performed in triplicate to control. * *p* < 0.05 vs. control; α *p* < 0.05 vs. single probiotic strains.

As illustrated in Figure 4A, all individual probiotic strains demonstrated the capacity to enhance cell viability in comparison to the control group (*p* < 0.05). Importantly, the combined formulation (MIX) further potentiated this effect, enhancing cell viability to a greater extent than the individual strains, with percentages of 44%, 78% and 54%, respectively, compared to *B. bifidum* novaBBF9, *L. fermentum* novaLF58 and *L. crispatus* novaLCR6 (*p* < 0.05). A similar pattern emerged for cell proliferation and cyclin D expression (Figure 4B,C). All strains positively influenced these parameters compared with untreated cells (*p* < 0.05). Furthermore, in this case, MIX was found to exert a greater beneficial effect than the individual probiotics by approximately 42% and 29% (vs. *B. bifidum* novaBBF9, *p* < 0.05), 60% and 74% (vs. *L. fermentum* novaLF58, *p* < 0.05) and 48.5% and 46% (vs. *L. crispatus* novaLCR6, *p* < 0.05). The findings indicate that metabolites secreted by probiotic strains in the basolateral compartment may positively influence myometrial cell viability and proliferation. Consequently, the impact of these treatments on ROS and TNF-α production was examined. The results for ROS production (Figure 4D) showed that all probiotic strains reduced ROS production below control levels (*p* < 0.05). Furthermore, the combined treatment further potentiated the impact of the individual probiotic strains, resulting in a reduction in ROS production by approximately 29% compared to *B. bifidum* novaBBF9 (*p* < 0.05), by approximately 49% compared to *L. fermentum* novaLF58 (*p* < 0.05) and by approximately 34% compared to *L. crispatus* novaLCR6 (*p* < 0.05). In relation to TNF-α production (Figure 4E), a comparable response was noted for all probiotic strains. A similar potentiation was observed with MIX, showing an approximate enhancement of 42% in the effect of the individual agents when compared with *B. bifidum* novaBBF9 (*p* < 0.05), by approximately 74% (vs. *L. fermentum* novaLF58, *p* < 0.05) and by approximately 53% (vs. *L. crispatus* novaLCR6, *p* < 0.05).

Overall, the metabolites released by the probiotic strains appear to improve trophism in myometrial cells, promoting vitality and proliferation while reducing oxidative stress and inflammation. The observed effect is significantly enhanced by the combined use of the strains, suggesting a synergistic action of the probiotic metabolites on the myometrial compartment.

**Figure 5:**
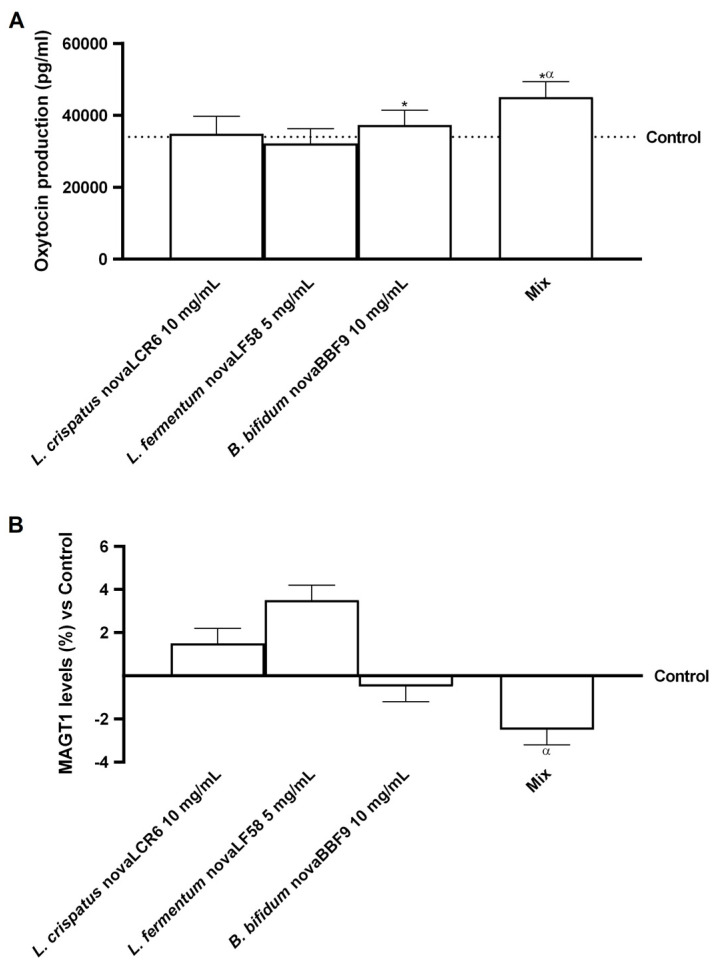
Contractile activity analysis in myometrial cells. In (**A**), Oxytocin production analysis was performed by a specific ELISA Kit; and in (**B**), MAGT1 levels analysis was measured by a specific ELISA kit. Mix = *L. crispatus* novaLCR6 10 mg/mL + *L. fermentum* novaLF58 5 mg/mL + *B. bifidum* novaBBF9 10 mg/mL. Data are expressed as mean ± SD (%) of 5 independent experiments performed in triplicate, normalised to the control. * *p* < 0.05 vs. control; α *p* < 0.05 vs. single probiotic strains.

Encouraged by these results, further experiments were conducted to investigate hormonal regulation. To date, several studies suggest that oxytocin may play a dual role: in the regulation of secretion of some anterior pituitary hormones and in the induction of myometrial contractility, independent of changes in intracellular calcium and magnesium concentrations in smooth muscle tissue [65]. Thus, to exclude myometrial hypercontraction, a factor negatively involved in myometrial homeostasis and thus in fertility, oxytocin and MAGT1 levels were measured. As reported in Figure 5A, oxytocin production in control cells (untreated cells) was measured at around 36,000 pg/mL. However, following treatment with *B. bifidum* novaBBF9, oxytocin production increased by approximately 10% compared to the control (*p* < 0.05), whereas the *L. crispatus* novaLCR6 strain showed oxytocin levels comparable to those of the control. In addition, *L. fermentum* novaLF58 caused a slight reduction in oxytocin levels of approximately 5% compared to the control (*p* < 0.05). Treatment with the combination of probiotics MIX resulted in a marked increase in oxytocin production, reaching values close to 45,000 pg/mL, corresponding to an increase of approximately 19% compared to the control (*p* < 0.05), by about 17% vs. *B. bifidum* novaBBF9 (*p* < 0.05), by about 29% vs. *L. fermentum* novaLF58 (*p* < 0.05) and by about 23% vs. *L. crispatus* novaLCR6 (*p* < 0.05).

Instead, the data shown in Figure 5B relate to the analysis of MAGT1 levels, i.e., the transporter responsible for maintaining intracellular magnesium levels. The percentage changes in MAGT1 levels compared to the control are shown. The results showed that all probiotic strains maintained MAGT1 levels at the same level as in the control, with no significant differences. In contrast, MIX was shown to induce a significant reduction in MAGT1 expression compared to individual probiotic strains, by about 80% vs. *B. bifidum* novaBBF9 (*p* < 0.05), about 2.4-fold vs. *L. fermentum* novaLF58 (*p* < 0.05) and about 1.6-fold vs. *L. crispatus* novaLCR6 (*p* < 0.05).

Overall, these results indicate that individual probiotic strains exert modest and sometimes divergent effects on oxytocin production and MAGT1 expression. In contrast, the combination of probiotic strains appears to have more pronounced and statistically significant effects, characterised by a significant increase in oxytocin production and a concomitant reduction in MAGT1 levels. These data support the hypothesis of a cooperative effect of probiotic strains, potentially relevant for the modulation of neuroendocrine mechanisms and cellular processes associated with magnesium homeostasis.

### 3.3. Analysis of the Effects of Probiotics on the Gut–Ovary Axis

To elucidate whether probiotic strains, administered individually or in combination, can also exert a positive influence on ovarian tissue, thereby enhancing its reproductive potential, the principal molecular mechanisms underlying ovarian dysfunction associated with infertility were analysed. In particular, the activation of the ERK and AKT signalling pathways was evaluated, given their fundamental roles in regulating proliferation, cell survival and hormonal responses in ovarian cells. Concurrently, the expression of PAK1, a downstream kinase of AKT involved in cytoskeletal remodelling and follicular competence, was analysed. Furthermore, the levels of ERβ and PAX8, two pivotal transcription factors in maintaining ovarian cell identity and regulating differentiation, were analysed. Consequently, the integrated analysis of these markers facilitates the delineation of the impact of probiotic treatments on intracellular pathways relevant to ovarian fertility. 

**Figure 6:**
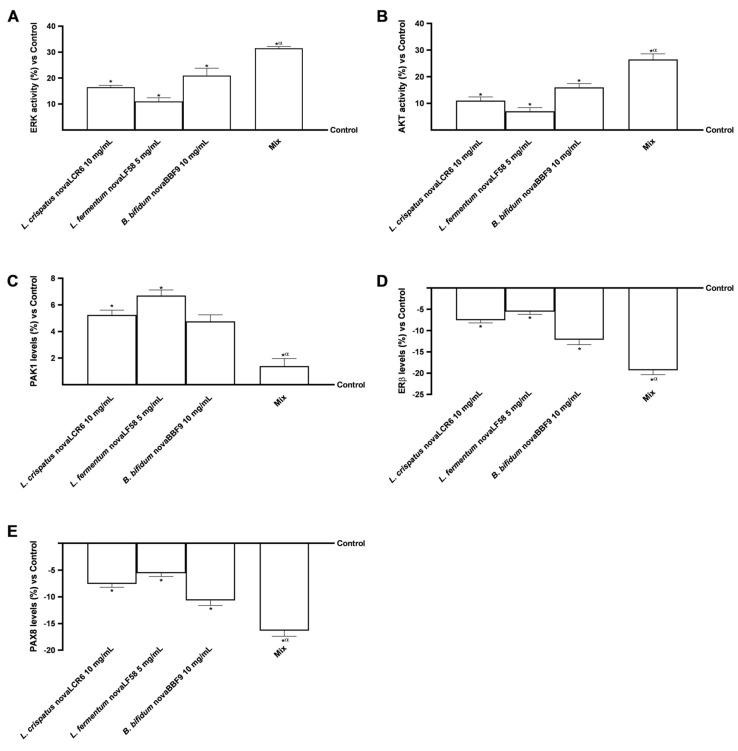
Intracellular pathway analysis in ovarian cells. In (**A**), ERK/MAPK activity was measured by specific assay kit; in (**B**), PI3K/AKT activity was measured by specific assay kit; in (**C**), PAK1 levels was determined by ELISA kit; in (**D**), Erβ levels was quantified by ELISA kit; and in (**E**), PAX8 levels was quantified by ELISA kit. Mix = *L. crispatus* novaLCR6 10 mg/mL + *L. fermentum* novaLF58 5 mg/mL + *B. bifidum* novaBBF9 10 mg/mL. Data are expressed as mean ± SD (%) of 5 independent experiments performed in triplicate, normalised to the control. * *p* < 0.05 vs. control; α *p* < 0.05 vs. single probiotic strains.

As indicated in Figure 6A, there was a significant increase in ERK activity in all treatment groups in comparison to the control group (*p* < 0.05). Specifically, *L. crispatus* novaLCR6 induced an increase of approximately 16.5% (*p* < 0.05), while *L. fermentum* novaLF58 caused a more modest increase of approximately 11% (*p* < 0.05), and *B. bifidum* novaBBF9 showed an intermediate increase of approximately 21% (*p* < 0.05). The combined formulation produced the strongest activation, reaching approximately 31.5% of control levels (*p* < 0.05). This increase was approximately 33% higher than that observed for *B. bifidum* novaBBF9 (*p* < 0.05), 65% higher than that for *L. fermentum* novaLF58 (*p* < 0.05) and 48% higher than that for *L. crispatus* novaLCR6 (*p* < 0.05), suggesting a cooperative effect.

A comparable trend was observed for AKT activity (Figure 6B). Indeed, *L. crispatus* novaLCR6 induced an increase of approximately 11%, *L. fermentum* novaLF58 an increase of approximately 7%, and *B. bifidum* novaBBF9 an increase of approximately 16%, in comparison with the control (*p* < 0.05). In line with ERK results, MIX increased AKT activity by approximately 26.5% in comparison to the control (*p* < 0.05), yielding a result that was approximately 40% higher than that of *B. bifidum* novaBBF9 (*p* < 0.05), 73.5% higher than that of *L. fermentum* novaLF58 (*p* < 0.05) and 58% higher than that of *L. crispatus* novaLCR6 (*p* < 0.05).

Concerning PAK1 (Figure 6C), the individual strains induced a significant increase in levels compared to the control, except *B. bifidum* novaBBF9 (*L. crispatus* novaLCR6 by approximately 5.25% and *L. fermentum* novaLF58 by 6.7%, *p* < 0.05). Conversely, the combined treatment maintained PAK1 at control levels and significantly reduced its expression by approximately 70.5%, 79% and 73% compared to *B. bifidum* novaBBF9, *L. fermentum* novaLF58 and *L. crispatus* novaLCR6, respectively (*p* < 0.05).

Finally, analysis of ERβ (Figure 6D) and PAX8 (Figure 6E) levels showed a significant reduction after treatment with probiotic strains alone compared to the control (*p* < 0.05). MIX demonstrated a greater reduction than the individual probiotic strains on both parameters, by approximately 37% and 35% vs. *B. bifidum* novaBBF9 (*p* < 0.05), approximately 71% and 66% vs. *L. fermentum* novaLF58 (*p* < 0.05) and approximately 61% and 54% vs. *L. crispatus* novaLCR6 (*p* < 0.05).

The data suggested that individual probiotic strains can significantly modulate signalling pathways and ovarian markers. However, MIX was found to exert a quantitatively superior and qualitatively more coordinated effect. The enhanced activation of ERK and AKT, accompanied by a concomitant reduction in PAK1, ERβ and PAX8, suggests a molecular environment conducive to improving ovarian function and reducing cellular mechanisms associated with infertility.

**Figure 7:**
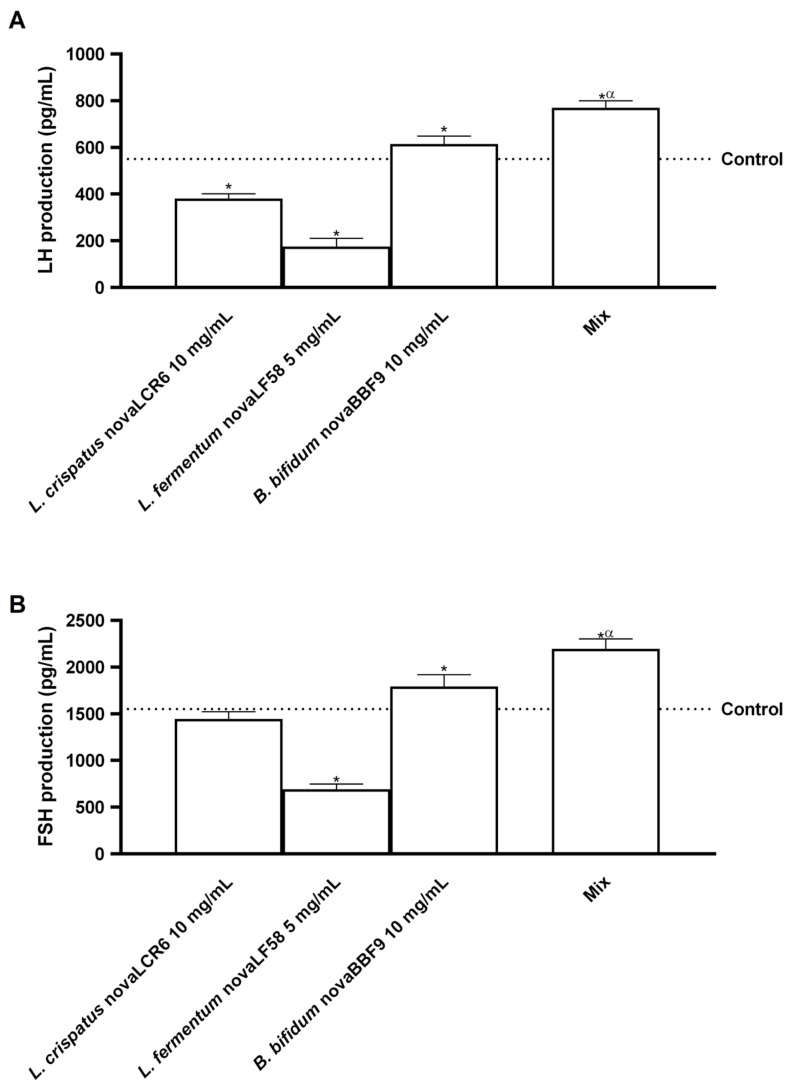
Hormonal activity analysis in ovarian cells. In (**A**), LH production was analysed by an ELISA kit, and in (**B**), FSH production was measured by an ELISA kit. Mix = *L. crispatus* novaLCR6 10 mg/mL + *L. fermentum* novaLF58 5 mg/mL + *B. bifidum* novaBBF9 10 mg/mL. Data are expressed as mean ± SD (%) of 5 independent experiments performed in triplicate, normalised to the control. * *p* < 0.05 vs. control; α *p* < 0.05 vs. single probiotic strains.

Finally, since LH and FSH play complementary roles in follicular development and ovulation through complex interactions in the hypothalamus, anterior pituitary gland, reproductive organs and oocytes, further experiments were conducted to analyse the effects of probiotics on ovarian cells. As shown in Figure 7A, among the individual probiotic strains, only *B. bifidum* novaBBF9 significantly increased LH production compared to the control (*p* < 0.05). Again, MIX was able to induce more pronounced effects, exceeding each strain by approximately 20% (vs. *B. bifidum* novaBBF9, *p* < 0.05), approximately 77% (vs. *L. fermentum*, *p* < 0.05) and approximately 50.5% (vs. *L. crispatus* novaLCR6, *p* < 0.05). A similar pattern of activity was observed for FSH production (Figure 7B); indeed, *B. bifidum* novaBBF9 induced higher FSH production than the control (*p* < 0.05), *L. fermentum* novaLF58 induced lower production than the control (*p* < 0.05), and *L. crispatus* novaLCR6 increased FSH levels to the same level as the control. As observed for FSH production, MIX again produced the strongest response, exceeding the effects of the individual strains by approximately 18.5%, 68.5% and 34% compared to *B. bifidum* novaBBF9, *L. fermentum* and *L. crispatus* novaLCR6, respectively (*p* < 0.05).

## 4. Discussion

Female fertility is conditioned to a multifaceted interplay among endocrine, metabolic, immunological and structural components of the reproductive system [66]. The success of conception and the maintenance of a pregnancy are contingent on the preservation of ovarian function and endocrine function, in addition to the functional integrity of uterine tissues, particularly the endometrium and myometrium [67]. Disruptions affecting any of these compartments can significantly impair reproductive potential. In this context, PCOS exemplifies a condition in which endocrine and metabolic dysfunctions coexist with immune dysregulation and intestinal dysbiosis, extending its pathophysiology beyond classical hormonal alterations [68].

Furthermore, the uterus’s function is pivotal to fertility. The myometrium, through coordinated contractile activity, is essential for sperm transport, embryo migration, and implantation. Alterations in myometrial trophism and contractility, frequently precipitated by inflammation, oxidative stress, or endocrine imbalance, have the potential to compromise uterine receptivity and adversely affect reproductive outcomes [69]. Recent evidence further highlights the intestinal microbiota as a key regulator of metabolic, endocrine and immune homeostasis, influencing both ovarian and uterine physiology. Intestinal dysbiosis has been associated with inflammation, altered sex hormone metabolism and insulin resistance. Conversely, a balanced microbiota that produces short-chain fatty acids has been shown to support reproductive function [70]. In this framework, our study investigated the molecular and cellular mechanisms by which a novel oral supplementation can exert a beneficial influence on myometrial and ovarian activities. Specifically, this study used a combination of probiotic strains (novaFERT^®^), namely *L. crispatus* novaLCR6, *L. fermentum* novaLF58 and *B. bifidum* novaBBF9, because systemic dysbiosis can destabilise uterine cell function, and because a healthy reproductive environment is typically defined by a *Lactobacillus*-dominant microbiome [70]. In the initial phase, intestinal analyses indicated that all individual strains at all selected concentrations enhanced cell viability, except for the highest concentration (50 mg/mL) of *L. fermentum* novaLF58, which did not show a positive effect compared with the control. However, for all strains, the optimal concentration was the lowest tested. The consistent observation that lower tested concentrations elicited greater epithelial viability supports the hypothesis of a hormetic-like response, whereby moderate microbial exposure optimally stimulates host signalling without inducing microenvironmental imbalance. Moreover, the combination of these probiotic strains resulted in a heightened effect on cell viability, intestinal monolayer integrity, probiotic adhesiveness and hydrophobicity, and SCFA production. SCFAs, such as butyric acid, that cross the intestinal barrier and reach the bloodstream act as second messengers in various tissues [26]. 

It should be acknowledged that Caco-2 cells originate from a human colorectal adenocarcinoma line; however, when cultured for 21 days, they differentiate into enterocyte-like monolayers expressing tight junction proteins and brush-border enzymes. This model is internationally validated by regulatory agencies (FDA and EMA) for predicting intestinal absorption and barrier function. During the initial *in vitro* experiments using Caco-2 cells assessed probiotic hydrophobicity and auto-aggregation. These assays were chosen not only to confirm the absence of cytotoxicity after MTT but also to provide insight into potential proliferative effects and adhesion capacity, which are critical for epithelial interaction and metabolite-mediated signaling [71]. These changes likely reflect adaptive responses to the host cell environment, where exposure to epithelial surfaces and cell-derived metabolites stimulates the expression of surface adhesins, extracellular polymeric substances, and other molecular components that promote bacterial aggregation and epithelial interaction. Enhanced hydrophobicity and aggregation are functionally significant, as they improve the ability of probiotic strains to adhere to the intestinal epithelium, form microcolonies, and persist within the local niche [71,72]. This increased colonization potential may facilitate more effective delivery of microbial metabolites, such as short-chain fatty acids, to downstream reproductive tissues, supporting signaling along the gut-myometrium and gut-ovary axes. Moreover, aggregation may confer additional protection to individual bacterial cells against environmental stressors, further sustaining their functional activity [73]. The observed enhancement in cell viability may be attributable to metabolites produced by the probiotics and modulate cell metabolism [74]. Enhanced cell viability observed at lower probiotic concentrations likely reflects a hormetic-like response, whereby moderate exposure to microbial metabolites, such as SCFA and lactic acid, optimally stimulates host signaling pathways without inducing cellular stress [75]. Hydrophobicity and aggregation data further support the strains’ potential for intestinal adhesion, a prerequisite for effective metabolite delivery along the gut–myometrium and gut–ovary axes [76,77].

Nevertheless, we recognise that this simplified in vitro system cannot fully replicate the structural and immunological complexity of the human intestinal mucosa. Therefore, the present findings at the intestinal level should be interpreted as mechanistic evidence supporting metabolite-mediated interactions rather than direct clinical extrapolation. These mechanistic findings provide a rationale for subsequent experiments on myometrial and ovarian cells, indicating that microbial metabolites and adhesion-mediated interactions could contribute to the observed modulation of cellular functions. While these results are derived from a simplified *in vitro* system, they offer valuable insight into possible gut-derived mechanisms influencing reproductive tissue physiology [76]. 

For this reason, in subsequent phases of experimentation, the effects of probiotics were analysed at the myometrial and ovarian levels following treatment with the probiotic combination, termed MIX, and individual probiotic strains. Indeed, the analysis in an in vitro intestine–myometrium axis, using Caco-2 and PHM1-41 cells, demonstrated that, while the MIX showed consistent and pronounced effects across most parameters, the responses to individual strains were variable and, in some cases, neutral or lower than control, indicating a parameter-dependent and strain-specific activity. These findings provide preliminary support for a potential gut–myometrial interaction. However, given the in vitro nature of the model, the results should be regarded as mechanistic and hypothesis-generating, indicating that the supplement’s active components may modulate myometrial cell activity rather than demonstrating a direct effect on uterine function in vivo [78]. These data were particularly notable following stimulation with the MIX, demonstrating the potential benefits of combining probiotic strains.

Reproductive health problems can also result from the interrelated processes of myometrial inflammation and ROS-induced oxidative stress. Although proper cellular activity depends on low, physiological amounts of ROS, excessive synthesis damages molecules and creates a persistently inflammatory environment by overwhelming the body’s antioxidant defences [79,80]. The substances under examination modulated both ROS production and inflammatory molecule production, demonstrating a protective effect. Specifically, MIX demonstrated the greatest efficacy in attenuating oxidative stress, significantly reducing ROS production and suppressing the inflammatory response, as measured by decreased TNF-α.

Another relevant aspect for uterine function is the impact on myometrial sensitivity. The myometrium is the primary target organ of oxytocin, the most potent uterotonic hormone, whose interaction with the oxytocin receptor is essential for inducing the uterine contractions necessary for gamete transport and childbirth [65]. Also, at the level of fundamental mechanisms, it is crucial to consider the role of the magnesium transporter MAGT1 and magnesium itself. Indeed, magnesium is an indispensable cofactor for enzymatic activity in biological systems, and it is a key muscle relaxant whose controlled release is vital for myometrial contractile activity [53]. Therefore, the positive modulation of oxytocin production and the effectiveness of probiotics and their combination in improving MAGT1 levels were of fundamental importance, suggesting that this formulation not only improves basic cellular health but also optimises the myometrium’s ability to respond to physiological contractile signals. This optimisation of responsiveness is essential to ensure appropriate uterine muscle tone and proper contractile function, which are fundamental to reproductive health.

Concurrently, the effects of the three probiotic strains, in isolation and in combination, on the gut–ovary axis were investigated. Consequently, the primary mechanisms implicated in ovarian dysfunction associated with female infertility were analysed along this axis. It has been suggested that the three probiotic strains exerted a positive modulatory effect on the ERK/MAPK and PI3K/AKT signalling pathways when administered individually. Moreover, the most significant outcomes were observed in response to treatment with MIX on both parameters, thereby corroborating the extant literature regarding the capacity of lactobacilli and bifidobacteria to release SCFAs that possess the ability to modulate ovarian tissue, stimulate follicular maturation and inhibit ovarian granulosa cell apoptosis by engaging PI3K/AKT-related signalling pathways [81,82]. These results are consistent with the mechanistic rationale from the intestinal assays, where metabolite production and adhesion properties suggest plausible pathways through which the probiotics could influence ovarian signalling and hormone secretion [76]. Another factor linked to ovarian health and function is ERβ expression; indeed, ERβ is highly expressed in ovarian granulosa cells and plays a critical role in follicle maturation, granulosa cell differentiation, and normal ovulatory function. Altered ERβ expression has been associated with ovarian dysfunction and impaired fertility [83]. Consequently, MIX’s capacity to considerably reduce ERβ levels may be pivotal in supporting normal ovarian physiology and maintaining healthy granulosa cell signalling.

Closely related to female reproductive health, PAX8 is a key player in ovarian physiology and pathology: it is a specific transcription factor, and its dysregulated expression has been implicated in epithelial transformation and ovarian carcinogenesis. Moderate PAX8 expression can be detected in a subset of normal ovarian surface epithelial cells and in the epithelium of inclusion cysts. In contrast, elevated PAX8 expression is characteristic of non-mucinous ovarian carcinomas, making it a useful marker for distinguishing neoplastic from non-neoplastic ovarian tissues [84]. In this context, the ability of MIX to significantly reduce PAX8 levels in ovarian cells may represent a beneficial modulation that supports physiological ovarian homeostasis by preventing aberrant transcriptional activation associated with pathological states. Similarly, PAK1 has been linked to ovarian cell function and pathology, where altered PAK1 levels are associated with changes in cytoskeletal dynamics and cell signalling that may contribute to abnormal proliferation and invasive behaviour in ovarian cells [85]. Therefore, the ability of MIX to maintain PAK1 at physiological levels, greater than that observed with single probiotic strains, highlights its potential importance in preserving normal signalling dynamics relevant to ovarian health and cellular function.

Finally, the modulation of ovarian hormones, particularly FSH and LH, was analysed. Impairment of gonadotropin production or action leads to relative or absolute LH and FSH deficiency, compromising gametogenesis and gonadal steroid production and thereby reducing fertility. In women, LH and FSH deficiency is a spectrum of conditions with different functional or organic causes, characterised by low or normal gonadotropin levels and low oestradiol levels. Reduced FSH and LH action is associated with reduced response to ovarian stimulation [86]. *B. bifidum* novaBBF9 alone was able to restore LH and FSH levels compared to the other probiotic strains *L. fermentum* novaLF58 and *L. crispatus* novaLCR6 alone. These effects were amplified when all three probiotic strains were used in combination and the data obtained suggested that such a combination could act on different and intertwined pathways.

In summary, the present study suggests that an orally administered, formulated supplement containing *L. crispatus* novaLCR6, *L. fermentum* novaLF58 and *B. bifidum* novaBBF9 may serve as a potent modulator of ovarian and myometrial cellular physiology, potentially acting through the gut–ovary and gut–myometrium axes. The observed biological effects of MIX may likely result from metabolic complementarity and increased epithelial niche saturation. While *Lactobacillus* strains contribute primarily to the lactic acid pool, *B. bifidum* novaBBF9 could expand the metabolic profile through acetate production, providing a broader range of ligands for protein G-coupled receptors (GPCR) signalling in distal reproductive tissues. This ‘metabolic combination’ may trigger a more robust homeostatic response in ovarian and myometrial cells than in individual strains, potentially through the simultaneous activation of multiple protective pathways and improved antioxidant capacity [86,87]. The findings provide preliminary mechanistic evidence supporting the potential of this probiotic formulation as a targeted intervention to enhance ovarian and myometrial homeostasis in vitro, which is a critical prerequisite for optimal reproductive function. Future investigations using factorial experimental designs and interaction modelling will be needed to further optimise strain ratios and quantitatively define synergistic interactions. Moreover, given the simplified nature of the model, these results should be interpreted with caution, and further validation in more physiologically integrated in vitro systems, animal models, and clinical studies will be necessary to establish their translational relevance.

## 5. Conclusions

The present study indicated that an oral supplement (novaFERT^®^) containing *L. crispatus* novaLCR6, *L. fermentum* novaLF58 and *B. bifidum* novaBBF9 exerted multiple modulatory effects on ovarian and myometrial cell physiology, likely mediated through the gut–ovary and gut–myometrial axes. The formulation improved the viability, proliferation and contractile function of myometrial cells, whilst simultaneously modulating oxidative stress and inflammatory responses. Furthermore, the supplement positively modulated key intracellular signalling pathways, including ERK/MAPK and PI3K/AKT, and preserved the physiological expression levels of critical markers such as ERβ, PAK1 and PAX8, suggesting its ability to support uterine homeostasis and maintain adequate hormonal responsiveness in ovarian cells. In conclusion, the available data provide robust in vitro preclinical evidence supporting the use of this targeted probiotic combination to enhance reproductive tissue function and potentially improve fertility outcomes. Further research is required to utilise physiologically integrated in vitro and in vivo models to comprehensively evaluate the translational relevance of these findings.

## Figures and Tables

**Table 1:** Sample concentration range.

Sample	Genus	Species	Strain	Low Dose (cfu)	Low Dose (mg)	High Dose (cfu)	High Dose (mg)
Probiotic	*Lactobacillus*	*crispatus*	novaLCR6 (DSM34348)	1 × 10^9^	10	5 × 10^9^	50
Probiotic	*Lactobacillus*	*fermentum*	novaLF58 (DSM 34340)	0.5 × 10^9^	5	5 × 10^9^	50
Probiotic	*Bifidobacterium*	*bifidum*	novaBBF9 (DSM 34337)	1 × 10^9^	10	5 × 10^9^	50

## Data Availability

The original contributions presented in the study are included in the article; further inquiries can be directed to the corresponding author.

## Abbreviations

The following abbreviations are used in this manuscript:

ATCC	American Type Culture Collection
CFU	Colony Forming Unit
DMEM	Dulbecco’s Modified Eagle’s Medium
DMEM-F12	Dulbecco’s Modified Eagle’s Medium/Nutrient F-12 Ham’s
EMA	European Medicines Agency
FAO	Food and Agriculture Organisation of the United Nations
FBS	Foetal Bovine Serum
FDA	The Food and Drug Administration
FSH	Follicle-Stimulating Hormone
LH	Luteinising Hormone
MAGT1	Magnesium Transport 1
NLRP3	NLR Family Pyrin Domain Containing 3
PBS	Phosphate-buffered saline
PCOS	Polycystic ovary syndrome
ROS	Radical oxygen species
SCFAs	Short-chain fatty acids
TEER	Transepithelial Electrical Resistance
TNF-α	Tumour Necrosis Factor α
TJ	Tight junction
WHO	World Health Organisation
